# High association of *Cryptosporidium* spp. infection with colon adenocarcinoma in Lebanese patients

**DOI:** 10.1371/journal.pone.0189422

**Published:** 2017-12-19

**Authors:** Marwan Osman, Sadia Benamrouz, Karine Guyot, Martha Baydoun, Emilie Frealle, Magali Chabe, Nausicaa Gantois, Baptiste Delaire, Anne Goffard, Albert Aoun, Nawaf Jurdi, Fouad Dabboussi, Gael Even, Christian Slomianny, Pierre Gosset, Monzer Hamze, Colette Creusy, Eric Viscogliosi, Gabriela Certad

**Affiliations:** 1 Institut Pasteur de Lille, Centre d’Infection et d’Immunité de Lille (CIIL), UMR CNRS 8204, INSERM U1019, Université de Lille, CHU de Lille, Biologie et Diversité des Pathogènes Eucaryotes Emergents (BDPEE), Lille, France; 2 Laboratoire Microbiologie Santé et Environnement (LMSE), Ecole Doctorale des Sciences et de Technologie, Faculté de Santé Publique, Université Libanaise, Tripoli, Lebanon; 3 Ecologie et Biodiversité, Faculté de Gestion, Economie et Sciences (FGES), Université Catholique de Lille, Lille, France; 4 BioMEMS, Université de Lille, CNRS, ISEN, UMR 8520 - IEMN, Lille, France; 5 BioGAP, groupe HEI-ISA-ISEN, Lille, France; 6 Centre Hospitalier Régional et Universitaire de Lille & Faculté de Médecine de Lille, Université Lille Nord de France, Laboratoire de Parasitologie-Mycologie, Centre de Biologie et Pathologie, Lille, France; 7 Faculté des sciences pharmaceutiques et biologiques, Université Lille Nord de France, Département de Parasitologie–Mycologie, Lille, France; 8 Service d’Anatomie et de Cytologie Pathologiques, Groupement des Hôpitaux de l’Université Catholique de Lille, Lille, France; 9 Molecular and Cellular Virology, University Lille, CNRS, INSERM, CHU Lille, Institut Pasteur de Lille, U1019-UMR 8204-CIIL-Centre d’Infection et d’Immunité de Lille, Lille, France; 10 Pathology Department, Faculty of Medicine, American University of Beirut, Beirut, Lebanon; 11 Genes Difussion, Douai, France-PEGASE-Biosciences, Lille, France; 12 Laboratory of Cell Physiology, INSERM U 1003, Université de Lille, Villeneuve d’Ascq, France; 13 Département de la Recherche Médicale, Groupement des Hôpitaux de l’Institut Catholique de Lille, Faculté de Médecine et Maïeutique, Université Catholique de Lille, Lille, France; "INSERM", FRANCE

## Abstract

**Background:**

The association between *Cryptosporidium* and human colon cancer has been reported in different populations. However, this association has not been well studied. In order to add new strong arguments for a probable link between cryptosporidiosis and colon human cancer, the aim of this study was to determine prevalence and to identify species of *Cryptosporidium* among Lebanese patients.

**Methodology and principal findings:**

Overall, 218 digestive biopsies were collected in Tripoli, Lebanon, from three groups of patients: (i) patients with recently diagnosed colon intraepithelial neoplasia/adenocarcinoma before any treatment (n = 72); (ii) patients with recently diagnosed stomach intraepithelial neoplasia/adenocarcinoma before any treatment (n = 21); and (iii) patients without digestive intraepithelial neoplasia/adenocarcinoma but with persistent digestive symptoms (n = 125). DNA extraction was performed from paraffin-embedded tissue. The presence of the parasite in tissues was confirmed by PCR, microscopic observation and immunofluorescence analysis. We identified a high rate (21%) of *Cryptosporidium* presence in biopsies from Lebanese patients with recently diagnosed colonic neoplasia/adenocarcinoma before any treatment. This prevalence was significantly higher compared to 7% of *Cryptosporidium* prevalence among patients without colon neoplasia but with persistent gastrointestinal symptoms (OR: 4, CI: 1.65–9.6, *P* = 0.001). When the comparison was done against normal biopsies, the risk of infection increased 11-fold in the group of patients with colon adenocarcinoma (OR: 11.315, CI: 1.44–89.02, *P* = 0.003).

**Conclusions:**

This is the first study performed in Lebanon reporting the prevalence of *Cryptosporidium* among patients with digestive cancer. These results show that *Cryptosporidium* is strongly associated with human colon cancer being maybe a potential etiological agent of this disease.

## Introduction

The protozoan *Cryptosporidium* constitutes a significant risk to humans and animals causing self-limiting diarrhea in immunocompetent hosts and life-threatening disease in immunocompromised hosts. Recently, a cohort study involving 22,500 children living in Africa and Asia revealed that *Cryptosporidium* represents one of the four pathogens responsible for severe diarrhea and mortality in infants and toddlers [1,2]. Additionally, our team has discovered that *Cryptosporidium parvum*, a species frequently isolated from humans and animals, is able to induce digestive adenocarcinoma in a rodent model, being the first time that an eukaryotic microorganism has been associated with neoplastic changes in the digestive epithelium of a mammalian host [3–6]. Consistently, some epidemiological studies have reported an association with cryptosporidiosis in patients with colorectal adenocarcinoma [7–9]. However, the correlation between cryptosporidiosis and human digestive cancer remains unclear at this time, and it is not known whether this intracellular parasite, considered an opportunistic agent, is able to induce gastrointestinal malignancies in humans. Therefore, these experimental and clinical observations represent a strong recommendation for further clinical research into the causal involvement of *Cryptosporidium* spp. in human digestive cancer. In order to add new arguments for a probable association between cryptosporidiosis and digestive human cancer, the main aim of this study was to determine prevalence and to identify species of *Cryptosporidium* among a Lebanese digestive cancer population. Interestingly, a high rate (21%) of *Cryptosporidium* presence was found in biopsies from Lebanese patients with recently diagnosed colonic neoplasia/adenocarcinoma before any treatment. These results provide new data about a potential role of this parasite in the development of colon adenocarcinoma. However, further studies are needed to reach definitive conclusions about this association.

## Materials and methods

### Ethics statement

This study was approved from the Lebanese Minister of Public Health (reference number: 4–39716). In accordance with the ethical standards of the Lebanese legislation physicians within the common clinical practice orally informed all adult patients about potential utilization of biopsies for research or secondary purpose, and participants manifested verbally their non-opposition. To obtain written consent was not possible due to the fact that this study was retrospective. However, attestation statements signed by the physicians recording the verbal non-opposition manifested by participants were obtained. Data were analysed anonymously.

### Study population and sample collection

Digestive biopsies fixed in formalin and embedded in paraffin were collected in Tripoli, Lebanon, between 2012 and 2013 from three groups of patients: (i) patients with recently diagnosed colon intraepithelial neoplasia/adenocarcinoma before any treatment (n = 72); (ii) patients with recently diagnosed stomach intraepithelial neoplasia/adenocarcinoma before any treatment (n = 21); and (iii) patients without colon or stomach intraepithelial neoplasia/adenocarcinoma but with persistent digestive symptoms that justified biopsy sampling (n = 125). For the purpose of statistical analysis the experimental group (i) was compared to different subgroups: a) all patients of groups (ii) and (iii) (n = 146). b) patients of group (iii) without colonic neoplasia but with other colonic pathology such as diverticulitis, colitis, polyps, inflammatory disease, etc. (n = 72); c) patients of group (iii) with a normal digestive biopsy (n = 44). In this group only patients without evidence of histological alterations were included. Finally group (i) was also compared to group (ii). The sample size corresponds to the number of samples that we could collect during a specific period due to logistic reasons. All patients were negative for human immunodeficiency virus.

### DNA extraction

Paraffined sections were treated with xylene and ethanol and then rehydrated. To disrupt the wall of potential oocysts, the samples were lysed using glass beads (Sigma Aldrich, Germany). DNA was extracted from 2 sections of 30 μm from each block using the QIAamp DNA Mini Kit (Qiagen GmbH, Hilden, Germany). The extracted DNA was quantified by a spectrophotometer by Nanodrop. In order to test fragmentation, 10 μl of DNA samples randomly selected were electrophoresed on a 1% agarose gel containing 0.5 μg/ml ethidium bromide. The gel was run at a low voltage and DNA ladders were finally visualized by a UV light. To avoid the risk of cross-contaminations between two consecutive paraffin blocks histological standard procedures for cleaning workspace and instruments were applied.

### Real-time PCR for *Cryptosporidium* spp. detection

The 18S rRNA real-time PCR was performed as previously described [10]. Positive PCR products were purified and sequenced by Genoscreen (Pasteur Institute, Lille, France). The sequences obtained were aligned using the BioEdit v7.0.1 package, and then compared with sequences of *Cryptosporidium* published in the NCBI. To avoid contamination of the histological samples standard procedures for cleaning workspace and instruments were applied including extensive microtome washing with DNA decontamination solution with each new sample section.

### Histological examination

*Cryptosporidium* was detected in histological sections after hematoxylin-eosin staining and immunofluorescence using a fluorescein isothiocyanate (FITC)-conjugated anti-*Cryptosporidium* spp. monoclonal antibody (Cellabs, Brookvale, New South Wales, Australia).

### Epstein-Barr virus detection

Genomic detection and quantification of Epstein-Barr virus (EBV) was performed from 10 μl of DNA using the quantitative EBV R-gene test system (Argene, BioMérieux Marcy l’Etoile, France).

### Statistical analysis

For the statistical analysis, Fisher’s exact test was used to test the relationship between different categorical variables. Odds ratios were calculated with *Cryptosporidium* infection as the main outcome using a logistic regression model.

A multivariate binary logistic regression analysis was used to test statistical relationship between *Cryptosporidium* infection and colon intraepithelial neoplasia/adenocarcinoma status taking in account confounding effect such as patient age, sex and affected organs. The general significance level was set at a *P*-value below 0.05. All analyses were performed using Vassarstats software and packages stats from the R statistical computing program [11].

## Results

**Fig 1:**
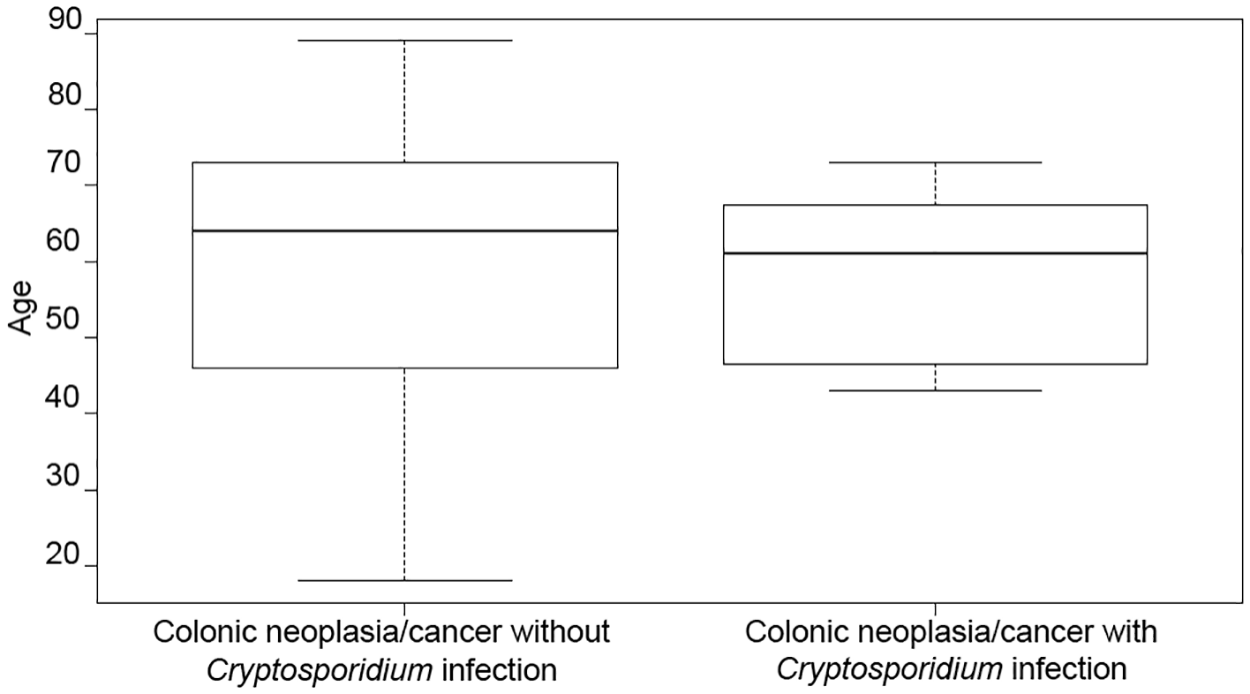
Age distribution among groups of patients with colonic neoplasia, infected or not by *Cryptosporidium*. Each box represents half of the data between upper and lower quartile, the black line being the median.

A total of 218 biopsies were collected in this study. The age of the patients was between 18 and 92 years (mean age: 50 ± 19). No significant differences related to age or sex were observed between groups. However, in patients with *Cryptosporidium* infection and colon neoplasia, the median age at time of diagnosis was lower compared to those patients with colon neoplasia without infection (X = 57,7 vs. X = 60.19). Additionally, the age distribution in these two groups differed considerably (Fig 1): for the second group, the age range was wider when compared to the first one, even if this difference was not significant. A possible explanation would be that the group control may include those persons with hereditary factors that predispose to get colon cancer before 30s but unfortunately, this kind of information was not available.

After DNA quantitation and quality assessment, using qPCR, *Cryptosporidium* infection was detected in 21% (15/72) of patients with recently diagnosed colon intraepithelial neoplasia/adenocarcinoma compared to 7% (9/146) of patients without digestive neoplasia but with persistent gastrointestinal symptoms, where the risk of *Cryptosporidium* infection was thus 4 times higher in the first group (OR: 4, CI: 1.65–9.6, *P* = 0.001). Similarly, when considering only colon disease, patients with colon intraepithelial neoplasia/adenocarcinoma also had a 4 times higher risk of *Cryptosporidium* infection than patients without neoplasia but with other colon pathologies (OR: 4.05, CI: 1.39–11.79, *P* = 0.006). When the comparison was done using normal biopsies, the risk of infection increased 11-fold in the group of patients with colon adenocarcinoma (OR: 11.315, CI: 1.44–89.02, *P* = 0.003). *Cryptosporidium* infection was found only in colonic but not in gastric neoplasia samples, and this difference was significant (*P* = 0.014) (Table 1).

**Table 1:** Frequency of *Cryptosporidium* infection in biopsies from patients with colonic intraepithelial neoplasia/adenocarcinoma compared to control groups.

Groups () and Subgroups []	Presence of *Cryptosporidium* N (%)	Absence of *Cryptosporidium* N (%)	OR	95% CI	*P*-value
Colonic intraepithelial neoplasia/adenocarcinoma (i)	15/72 (20.83)	57/72 (79.16)	4.006	(1.65–9.679)	0.0016*
Other digestive pathologies [a]	9/146 (6.16)	137/146 (93.83)			
Colonic intraepithelial neoplasia/adenocarcinoma (i)	15/72 (20.83)	57/72 (79.16)	4.05	(1.39–11.79)	0.0062*
Other colonic pathologies (without intraepithelial neoplasia/adenocarcinoma) [b]	5/82 (6.09)	77/82 (93.90)			
Colonic intraepithelial neoplasia/adenocarcinoma (i)	15/72 (20.83)	57/72 (79.16)	11.315	(1.44–89.02)	0.0031*
Normal digestive biopsies [c]	1/44 (2.27)	43/44 (97.72)			
Colonic intraepithelial neoplasia/adenocarcinoma (i)	15/72 (20.83)	57/72 (79.16)	NA	NA	0.01*
Gastric intraepithelial neoplasia/adenocarcinoma (ii)	0/21 (0)	21/21(100)			

NA/Not applicable.

(i) patients with recently diagnosed colon intraepithelial neoplasia/adenocarcinoma before any treatment (n = 72); (ii) patients with recently diagnosed stomach intraepithelial neoplasia/adenocarcinoma before any treatment (n = 21); and (iii) patients without colon or stomach intraepithelial neoplasia/adenocarcinoma but with persistent digestive symptoms that justified biopsy sampling (n = 125). For the purpose of statistical analysis the experimental group (i) was compared to different subgroups: a) all patients of groups (ii) and (iii) (n = 146). b) patients of group (iii) without colonic neoplasia but with other colonic pathology such as diverticulitis, colitis, polyps, inflammatory disease, etc. (n = 72); c) patients of group (iii) with a normal digestive biopsy (n = 44). In this group only patients without evidence of histological alterations were included. Finally group (i) was also compared to group (ii).

* Significant *P*-value.

Additionally, we checked that positive cases did not involve consecutive samples and the presence of the parasite in tissues was verified by microscopic observation.

**Fig 2:**
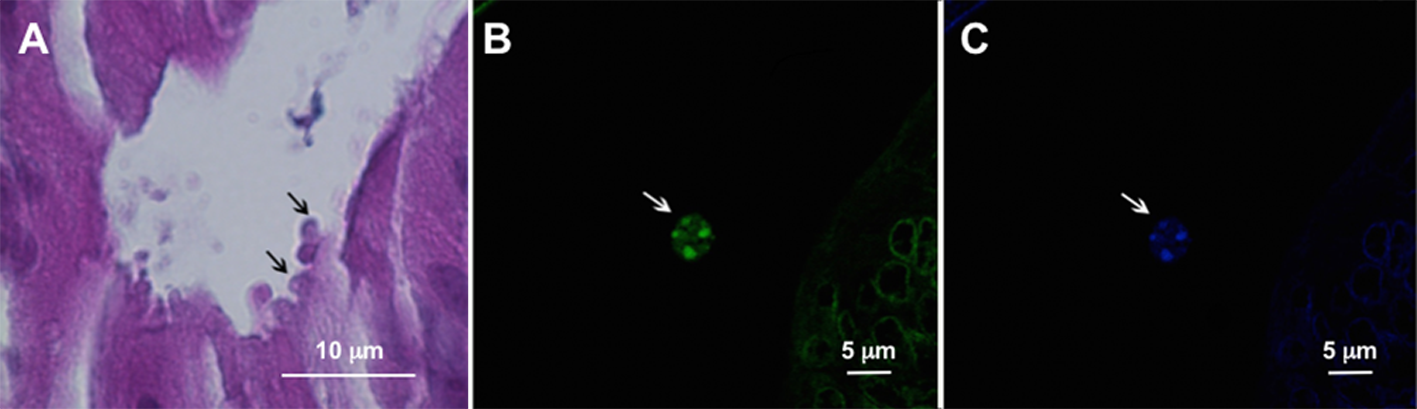
Histological sections from a colon adenocarcinoma associated to *Cryptosporidium* infection. (A) *Cryptosporidium* developmental stages were observed in the apical position (arrows) within the epithelial cells of the intestinal glands (hematoxylin and eosin). (B) A *Cryptosporidium* oocyst is shown (arrow) in the lumen of an intestinal gland after immunofluorescence (fluorescein isothiocyanate (FITC)-conjugated anti-*Cryptosporidium* spp. MAbs.) (C) Four sporozoites in the oocyst were observed (arrow) (staining with 4,6-diamidino 2-phenylindole dihydrochloride (DAPI)).

The multivariate binary logistic regression analysis showed that the variable colonic neoplasia/cancer continued to be significantly associated to *Cryptosporidium* infection after adjusting by age, sex or affected organ (Table 2). Inflammation was not significantly associated with the presence of the parasite (*P* = 0.3). After genotyping *Cryptosporidium* positive cases, two *Cryptosporidium* species were found: 19 (79.2%) samples were identified as *C*. *hominis*, while 5 (20.8%) were identified as *C*. *parvum*. When only colon intraepithelial neoplasia/adenocarcinoma was considered, this proportion was maintained: the presence of 7 (78%) *C*. *hominis* and 2 (22%) *C*. *parvum* isolates was confirmed. The sequences obtained showed 100% identity with GenBank reference sequences. There were not differences in the risk for colon cancer when these species were considered independently. The presence of the parasites in tissues was confirmed by microscopic observation in 11 colonic neoplastic sections out of 15 (Fig 2).

**Table 2:** Multivariate binary logistic regression analysis of the association between *Cryptosporidium* infection and colonic neoplasia/cancer development, age, sex or affected organ.

Variables	Estimate	Std. Error	Z value	*P*-value
Colonic neoplasia/ cancer	1.06350	0.50478	2.107	0.0351*
Age	-0.01439	0.01307	-1.101	0.2709
Sex	-0.00817	0.45139	-0.018	0.9856
Affected organ	-0.67756	0.58021	-1.168	0.2429

* Significant *P*-value.

In order to exclude EBV, an oncogenic pathogen potentially responsible for colon cancer, biopsies from cancerous and control subjects were analyzed. Results showed a homogeneous distribution of the virus among groups of *Cryptosporidium*-infected and non-infected patients, as well as among subjects with and without colon cancer.

## Discussion

The data herein indicates that *Cryptosporidium* was highly prevalent among Lebanese patients with colon intraepithelial neoplasia/adenocarcinoma, and this prevalence was significantly higher compared with the burden of cryptosporidiosis in control groups.

This prevalence of *Cryptosporidium* detected in these patients is also higher compared with prevalence reported in previous Lebanese epidemiological studies: 10% in *Cryptosporidium*-symptomatic patients and schoolchildren, and 5% detected in the general population [12,13], while the prevalence in the control group was in the range previously reported in the general Lebanese population [12].

Our results also showed a homogeneous distribution of other potential oncogenic pathogen, such as EBV [14] among groups, consistent with the fact that this virus infects almost everyone in early life and persists throughout life.

On the other hand, the molecular characterization of *Cryptosporidium* isolates allowed the identification of either *C*. *hominis* or *C*. *parvum*, with the former being predominant (79%). These results confirm recent reports in Lebanon showing the presence of these two species with the predominance of *C*. *hominis* [12]. Furthermore, for the first time, *C*. *hominis* has been associated with colon adenocarcinoma.

Because *Cryptosporidium* is an opportunistic agent that causes significant morbidity and mortality in immunocompromised patients, it is possible that individuals with malignancies, have a higher risk of developing infection with this parasite, especially when their immunosuppression is more severe. In this study, samples originated from recently diagnosed HIV negative patients, and before any treatment to avoid the decline in CD4+ and CD8+ T-cell counts. Then, the risk of an opportunistic infection due to the presence of cancer as immunodebilitating condition seemed to be low. However, one of the limitations of this study was the lack of information concerning the CD4+ and CD8+ counts of these patients. Further studies should be done taking into account this variable.

Associations between *Cryptosporidium* infection and digestive neoplasia in different populations have been described. A recent study conveyed that the risk of developing a colon carcinoma is significantly elevated among AIDS patients presenting cryptosporidiosis [9]. A possible association between human cryptosporidiosis and bile-duct carcinoma was suggested in children with X-linked hyper-IgM syndrome [15].

Furthermore, epidemiological studies in Poland reported a frequency of 18% and 12.6% of cryptosporidiosis in patients with recently diagnosed colorectal cancer before any immunosuppressive treatment, but in these studies the species responsible for infection were not determined [7, 8].

In fact, the potential role of *Cryptosporidium* in the development of neoplasia would not be surprising, considering that epidemiological and clinical reports indicate that eukaryotic protozoan, such as intracellular apicomplexan that cause diseases of medical or economic importance, can be linked to various cancers, for instance: *Theileria* induce host cell transformation while *Plasmodium* was linked epidemiologically to the "African lymphoma belt" over fifty years ago [16]. These intracellular eukaryotic parasites hijack cellular pathways to manipulate the host cell epigenome, cellular machinery, signaling pathways and epigenetic programs and marks, such as methylation and acetylation, for their own benefit [16].

Particularly, it has been reported that *C*. *parvum* for its survival and transmission interferes with host signaling pathways [17, 18]. For instance, in an *in vitro* model of biliary cryptosporidiosis *C*. *parvum* was shown to activate the NF-κB pathway in infected cells, preventing the induction of apoptosis after infection [19]. Interestingly, NF-κB family of transcription factors regulates the activation of several intracellular survival signals including the c-Myc protooncogene. Indeed, activation of NF-κB has been observed in many cancers, including colon cancer [20]. However, it has been found in *in vitro* studies that *C*. *parvum*, can modulate host-cell apoptosis, inhibiting apoptosis at the trophozoite stage and promoting this process at the sporozoite and merozoite stages [21]. Modulation of apoptotic pathways was also investigated by microarray analysis in an *in vitro* model using human ileocaecal HCT8 cells. Genome wide expression profiling revealed high proportion of apoptosis genes regulated during *C*. *parvum* infection [22]. In fact, resistance to apoptosis could be a crucial step in the progression to malignancy [17].

Moreover, it is well known that the parasite induces modifications of the host actin cytoskeleton of intestinal epithelial cells although little information is available about the significance of the host actin remodelling process. Several studies reported that infection of intestinal and biliary epithelial cells requires host cell actin polymerization and cytoskeleton remodelling [18]. This polymerization uses the actin branching and nucleation machinery of the Arp2/3complex of proteins. Several signaling axes such as those of PI3-kinase, guanine exchange factor, Frabin-dependent activation of the small GTPase, and CDC42- and c-Src-dependent activation of cortactin have been identified to modulate actin reorganization and *Cryptosporidium* internalization [18]. In consistence, recently it was found hat *C*. *parvum*, independently of the strain, is able to modulate host cytoskeleton activities and several host-cell biological processes via the Wnt signaling pathway in SCID mice [23].

## Conclusions

This is the first study performed in Lebanon reporting the prevalence of *Cryptosporidium* among patients with digestive cancer. These results show that *Cryptosporidium* is strongly associated with human colon cancer providing new data about a potential role of this parasite in the development of colon adenocarcinoma. However, further studies are needed to reach definitive conclusions about this association. The last but not least, changes in bowel habits, including diarrhea, have been found as significant predictors of colorectal cancer. Maybe presence of *Cryptosporidium* infection could be one of the causes explaining these symptoms.

Research into this topic would be worthwhile, since the incidence of *Cryptosporidium* infection seems to be increasing worldwide [1,2,13,24]. In addition, the World Health Organization acknowledges that nowadays 20% of cancers are due to infectious agents, and some authors have hypothesized that within 2050 the great majority of cancers will be considered to have an infectious origin [25].
